# Can we learn to manage stress? A randomized controlled trial carried out on university students

**DOI:** 10.1371/journal.pone.0200997

**Published:** 2018-09-05

**Authors:** Dalia Saleh, Nathalie Camart, Fouad Sbeira, Lucia Romo

**Affiliations:** 1 EA4430 CLIPSYD, UFR SPSE, Paris Nanterre University, Nanterre, France; 2 Counseling Psychology, Tishreen University, Latakia, Syria; 3 CMME, Centre Hospitalier Sainte Anne, Unité Inserm U864, CPN, Paris, France; TNO, NETHERLANDS

## Abstract

In our research, we examined the efficacy of an Internet-based stress management program. Our interest in evaluating this type of intervention is based on the increasing accessibility of the Internet today, the growth of Internet-based interventions for various psychopathological problems, and the observation that despite the prevalence of stress among university students, only a fraction of students ever seek professional help. Methodology: “I’m managing my stress” *(“Je gère mon stresse”)*, an Internet-based self-help program composed of four sessions, was examined in this study. The aforementioned program is based on cognitive-behavioral therapy and was inspired by the “*Funambule*” program in Quebec. Four questionnaires (Perceived Stress Scale, Rosenberg Self-Esteem Scale, Scale of Satisfaction in Studies, and General Health Questionnaire) uploaded online were answered thrice: during “preintervention”, “postintervention”, and “follow-up” stages, the latter of which occurred three months after the intervention. The sample comprised 128 university students, with the majority being women (81.25%). The subjects were divided randomly into two groups (an experimental group and a control group that did not follow the program). Results: The self-esteem scores of the control group were significantly higher than those of the experimental group at the preintervention stage, but this difference disappeared at the postintervention and follow-up stages. There were also significantly lower scores on the General Health Questionnaire subfactors of somatic symptoms and anxiety/insomnia in the experimental group than in the control group during the postintervention stage, though no differences were observed before the intervention. These differences no longer remained after three months. ANOVA revealed significant effects of the intervention over time in the experimental group. Effects were observed at both the postintervention and follow-up stages for self-esteem, perceived stress, satisfaction in studies, and in the somatic symptoms, anxiety and insomnia and severe depression aspects of the General Health Questionnaire (Cohen’s d = 0.38 to 4.58). In contrast, no effects were observed in the control group. Conclusion: This type of Internet-based program has the ability to reach a large number of students due to its rather short format and accessibility. It has already shown improvements in terms of the levels of perceived stress, psychological distress and satisfaction with studies. The option of online interventions could appeal specifically to students who do not seek professional help. However, even though these results are promising at the postintervention stage, they are limited, as indicated by the lack of significant differences between the two groups after the initial three months of follow-up. We still, specifically, need to improve this intervention program and, generally, need more research to address the methodological problems raised by this type of intervention.

**Trial registration**: ISRCTN registry, ISRCTN13709272

## Introduction

University students are a category of people who are particularly vulnerable to stress [1–3] and present, according to scientific literature, high levels of stress [4–9]. They are prone to stress-related issues such as anxiety, depression [10–12], eating disorders [13–15], consumption of psychoactive substances [16,17], and sleep disorders [12,18,19]. The rates of psychological morbidity among university students are higher than those seen in the general population [10,11,20–24].

According to the literature, 83% of university students feel tired [25], 60% have low self-esteem [26] and 15% harbor suicidal thoughts [27]. In fact, the prevalence of several mental health issues among university students is always high, regardless of the issue. For example, the mental health distress rate ranges from 21% to 82% [26,28–30], the depression rate between 13% and 53% [31–34], anxiety between 34% and 47% [34–36], and stress between 33% and 79% [8,23,37,38]. More than half of students suffer from at least one mental health problem according to a study undertaken by Zivin and his team. In their study, 50% of students who declared having mental health issues, such as depression, anxiety and suicidal thoughts, did not seek help [39].

The link between stress and psychological distress has been theoretically presented in Lazarus and Folkman’s transactional model in 1984 [4]. Their work is often applied in stress-management programs [40].

Finding efficient strategies to reinforce the feeling of personal efficacy and the efficient management of difficulties in certain groups such as students are essential to help these groups adapt to challenges and maintain a good standard of living [41]. Different types of interventions have already proven their efficacy [42–44].

The Internet has become an essential tool in the field of “self-help” interventions in mental health. These interventions can be defined by the fact that they allow online access to a therapeutic-aimed program [45]. This type of intervention via the Internet has several goals, such as reducing the risks leading to targeted problems [45], such as the levels of stress, anxiety and depression [46]; raising the level of well-being [47]; enhancing adaptation strategies [48]; and even increasing weekly physical activity [49]. The advantages of these online trials are their accessibility, continuous availability, confidentiality, and discretion, especially for people who do not want to seek medical help in a health center, as well as the opportunity to extend the program to a large population in an economical way [45].

Online application studies in this field are being developed to treat several problems, such as panic disorder [50], depression [51–53], anxiety [54–56], insomnia [57], posttraumatic stress [58], alcohol abuse [59], binge drinking [60], social phobia [61,62], and behavioral problems [63]. Stress management is one of the applications that has been suggested for diverse population groups [64–67].

Online stress management interventions aim to manage stress as well as other psychopathological issues [45] such as anxiety [68,69], prevention of obesity [70] and psychological distress [71]. In our literature review, according to a meta-analysis conducted in 2016 [45], only six studies had stress management as their principal goal [46–49,72], and only three of these studies were carried out with university students [45,46,48,49].

Despite its clear efficacy and numerous advantages [46,48], this type of intervention for stress management has its limits [73] and a major methodological problem, that is, a high attrition rate [45].

The aim of the current research is to measure the efficacy of an online stress management program for university students based on several mental health variables: self-esteem, perceived stress and its two subfactors (perceived helplessness and perceived self-efficacy), psychological distress and its four subfactors (somatic symptoms, anxiety/insomnia, social dysfunction, severe depression) and satisfaction with studies.

## Materials and methods

### Design of study

**Fig 1:**
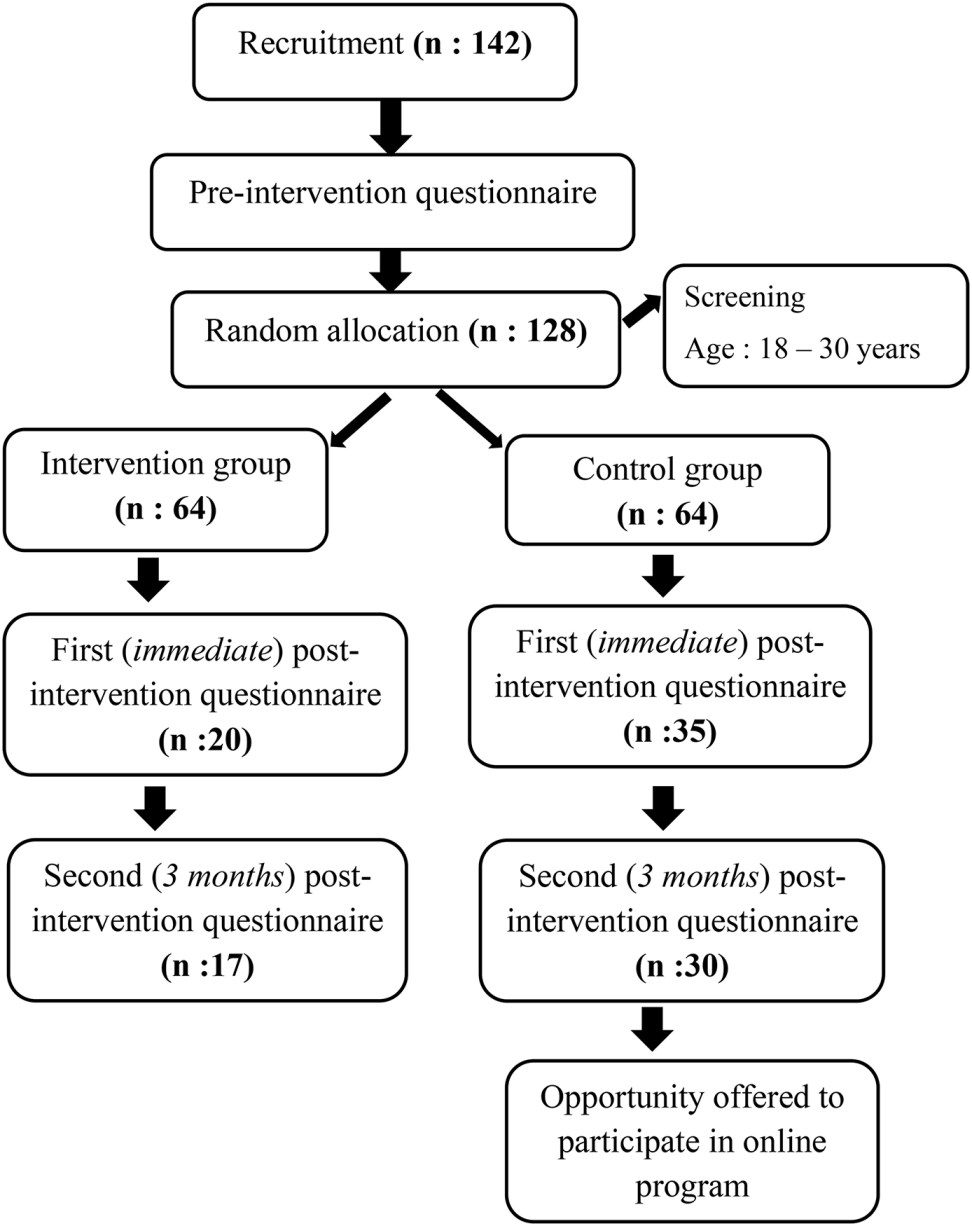
Trial schema.

This protocol used a randomized controlled trial to determine the efficacy of an online stress management intervention for university students (Fig 1). The inclusion criteria included the following: being a student at a French university, having mastered the French language, being aged between 18 and 30, and having an e-mail address and access to the Internet.

The subjects were randomly allocated into one of the two groups: an experimental group (those who followed the program) and a control group (those who did not follow the program). The subjects in the control group were informed that they had been randomly assigned to a waiting group, that they would not be following the program during the study, and that they could follow it afterwards if they so wished. The study followed the CONSORT 2010 Statement’s advice about randomized controlled trials [74].

### Participants

A total of 142 students voluntarily signed up for baseline assessments, and 16 participants were excluded (aged under 18 or over 30). The sample consisted of 128 university students (64 in the experimental group and 64 in the control group), with a female majority (81.25% in the whole sample, 82.81% in the experimental group and 79.69% in the control group). The mean age of the whole sample was 22.54 years (Standard Deviation or SD: 3), with no difference between the two groups in terms of age. The students were from all academic years, from the first year at university to PhD-level, and from different study programs: Philosophy, Languages, Literature, Economy, Management, Mathematics, Computing, Psychology, Law and Political Science, Medicine, etc. (Table 1).

**Table 1:** Participant characteristics n (%).

Participants’ characteristics		Percentage frequency			*p*
		Total (n = 128	Group 1 (n = 64	Group 2 (n = 64	
**Gender**	Women	104 (81.2)	(82.8)	(79.6)	0.6
	Men	24 (18.7)	(17.1)	(20.3)	
**Home university**	Paris Nanterre La Defense	99(77.3)	(79.6)	(75)	0.5
**Year of studies**	L1: First academic year	17(13.2)	(15.6)	(10.9)	0.5
	L2: Second academic year	15 (11.7)	(14)	(9.3)	
	L3: Third academic year	33(25.7)	(21.8)	(29.6)	
	M1: First year of Master’s degree	31(24.2)	(28.1)	(20.3)	
	M2: Second year of Master’s degree	18 (14)	(12.5)	(15.6)	
	PhD	14(10.9)	(7.8)	(14)	
**Academic sector according to UFR classification**	Foreign cultures and languages (LCE)	2.3(2.3)	(3.1)	(1.5)	0.1
	Philosophy, Information-Communication, Language, Literature, Performing Arts (PHILLIA)	9 (7)	(10.9)	(3.1)	
	Economics, Management, Mathematics, Computer Science (SEGMI)	7 (5.4)	(1.5)	(9.3)	
	Law and Political Science (DSP)	23(17.9)	(18.7)	(17.1)	
	Psychological Sciences and Educational Sciences (SPSE)	41(32)	(35.9)	(28.1)	
	Social Sciences and Administration (SSA)	12(9.3)	(9.3)	(9.3)	
	Other	33(25.7)	(20.3)	(31.2)	
**Repetition of academic year**	No	89(69.5)	(75)	(64)	0.2

Group 1 = experimental group; group 2 = control group. P<0.05 using Student’s t-test or chi-squared test.

### Recruitment and procedure

The research was presented as an assessment of a stress management intervention carried out with university students, whose participation was anonymous and voluntary. The recruitment occurred mostly on the Paris Nanterre University website, on which an advertisement for research was published. This allowed publicity in the student newsletter and on the university’s social media outlets. Posters were also put up in the campus and on social media.

### Stages of research

The research occurred between November 2015 and June 2016.

Preintervention (baseline/October 2015-January 2016): After providing their consent, the subjects received an Internet link to take part in the research online. Gift vouchers were offered to all participants as an incentive. Intervention “experimental group”/“control group”: In January, the students who were allocated into the experimental group were informed of their group assignment. They then provided their consent to participate in the intervention and perform tasks between sessions. They were invited to visit the website once a week and to spend at least 20 minutes on it per visit. They could visit the page for longer or more frequently if they wished. At the same time, the subjects in the control group were informed that they had been randomly allocated into the control group and that they could follow the program afterwards if they so wished. Postintervention assessment (February 2016): Just after the end of the intervention in March, the participants of both groups were simultaneously invited to answer the battery of questionnaires by email for the first “postassessment”. The "follow-up" (second postassessment) occurred three months later for both groups, in June. Reminder emails were sent in cases of no response.

### Intervention (« *I’m managing my stress »*) (February 2016)

We created an online stress management pilot program entitled “*I’m managing my stress”*. It was inspired by the “*Funambule”* program for teenagers developed by Dumont and his team [40] in Canada, in which cognitive-behavioral therapy techniques are used over 8 sessions.

The **“Funambule, for balanced stress management” program**, developed by Dumont and his team [40,75], is an intervention program designed to help young people aged 12 to 18 manage their stress better. It is undertaken in 8 weekly sessions of at least an hour and a half each. The content of the sessions is organized into 4 parts to target 4 goals: the perception of stress, the body, thoughts, and adaptation strategies [40,75]. The conception of the program was inspired by the transactional theory of stress developed by Lazarus and Folkmans in 1984, which aimed to strengthen protective resources [76]. Beneficial effects and a significant improvement of stress management in the experimental group were noted for participants using this program [75].

The “I’m managing my stress” program that we developed has the same objectives and aims, but as it is a pilot program inspired by “Funambule”, we therefore reworked and adapted it to fit the Internet and our study population (adults, not children as in the original program). The program consists of four sessions, each 20 minutes long, including psycho-education, practical exercises and one to two weekly activities that the participant is asked to complete (prescription of tasks, as is customary in cognitive-behavioral techniques). The goal is for the students to learn easy techniques to help them handle stressful situations better.

The first session is “psycho-education”, with information for the participants on how to identify and understand stress, measure stress levels, and determine stress sources. The second session aims to develop body relaxation using practical exercises adapted for the Internet, such as a contraction and relaxation method or diaphragmatic breathing. The third session is based on cognitive-behavioral techniques, such as Beck’s three columns, the Meichenbaum method, and problem solving. The objective of this session is to develop positive thinking [40]. The fourth session focuses on adaptation strategies such as time management, exam preparation, and the use of DIY anti-stress cards [40,75]. The final session is used to collect the participants’ opinions on the program in order to assess the program.

This program is interactive to the extent that the participants are invited to provide comments and opinions after each exercise and at the end of each session, at every stage of the program.

An email was sent to invite the participants at the start of each session to go online if they had not visited the website spontaneously; the first reminder was sent within three days after the start of the sessions. The students could contact us any time via a specific email address with any questions or complementary information on the program. Responses were sent within 48 hours.

### Measures

We split the questionnaires used in our study into two sections. First, the students provided socio-demographic data concerning gender, age, place of residence, current year of study, study program and university of origin. The second part was a collection of questionnaires, including four uploaded ones during the preintervention, postintervention, and follow-up (3 months after the intervention) stages.

- Stress was assessed with the Perceived Stress Scale (PSS-10) using the 10-item version. This method was established by Cohen and his team [77] It was subsequently translated and validated in French by Bellinghausen and his team. Using a 5-point scale, from 1 (which means “never”) to 5 (“very often”), each variable’s frequency is rated. This scale includes two subfactors: perceived helplessness and perceived self-efficacy [78]. Two thresholds were decided upon: a score over 24 indicated anxiety, and a score over 26 indicated depression [79]. The Cronbach’s alpha coefficient calculated for this assessment was (.84).
- To measure self-esteem, we decided to use a 10-item version of the Rosenberg Self-Esteem Scale (RSES), which was established by Morris Rosenberg [80] and then later translated and validated in French by Vallieres and his team. The participants were invited to use a 4-point Likert scale format, with their answers to the items ranging from “strongly agree” to “strongly disagree” [81]. Low self-esteem was indicated by a score under 30 [82] In this study, Cronbach’s alpha coefficient was (.9).
- To evaluate the participants’ satisfaction in their studies, we opted for the 5-item version of the ESDE, developed by Bissonnette and Vallerand, which was translated into French and validated by Vallerand and his team. In this assessment, five items are answered on a scale that ranges from 1 (“strongly disagree”) to 7 (“strongly agree”) [83]. The higher the score, the higher the participant’s satisfaction in their studies [84]. In this study, Cronbach’s alpha coefficient was (.79).
- We measured psychological distress using the 28-item General Health Questionnaire (GHQ-28), a questionnaire developed by Goldberg [85] and then translated and validated in French by Bolognini and his team [86]. It consists of 4 subscales that evaluate somatic symptoms, anxiety/insomnia, social dysfunction and severe depression [85]. In each of these sections, participants are invited to answer 7 items using a 4-point Likert scale [86]. Psychological distress is indicated by a score greater than or equal to 5 [87]. In this study, Cronbach’s alpha coefficient was (.65).

### Ethics

The ethics committee of the Psychological Science and Learning Science Department at the University of Paris Ouest Nanterre La Défense, UFR SPE (Department of Psychology and Education) in May 2015 and the CNIL (National Commission for Computing and Freedom) in November 2014 [n°: 1811031 v 0] gave their approval for our study protocol. We also obtained written consent from each student prior to their participation, as required by the Helsinki Declaration.

The research team obtained written permission from the creators of “Funambule”, giving us the right to take inspiration from their program to create an intervention program adapted to the Internet. A report on the study was sent to the creators of “Funambule” after each stage.

The authors confirm that all ongoing and related trials for this intervention are registered.

### Feasibility study

The feasibility of the study was tested in a pilot study between January and March 2015, in which a first group of 18 students (mean age = 23.64, SD = 5.62) underwent an intervention.

During the feasibility study, we noted a high level of attrition, meaning a loss of participants over time. In response to this problem, we decided to offer an incentive in the form of gift vouchers to all participants who fully participated in the research [88].

This approach was based on the results of a study by Fridric and his team [89], who encountered this problem in similar experiments. The participants’ comments also allowed us to improve our program’s form.

### Statistics

To allocate the participants into two groups (intervention/experimental and control), we used SPSS (IBM v. 23) random sampling software.

A standard descriptive analysis assessed sample characteristics (in general and by group), and Student’s t-tests and chi-squared test were used to measure the homogeneity between the two groups. To compare the two groups (intervention and control) at each stage (T1 = preassessment, T2 = postassessment and T3 = follow-up), we used the Mann-Whitney U test because the variables: *self-esteem*, *life satisfaction in studies*, *perceived stress and its two sub factors (perceived helplessness and perceived self-efficacy)*, *psychological distress and its 4 sub factors (somatic symptoms*, *anxiety/insomnia*, *social dysfunction*, *severe depression)*, did not follow a normal distribution.

To examine intra-group changes in the mental health variables’ scores studied in our research, that is, self-esteem, life satisfaction in studies, perceived stress and its two subfactors (perceived helplessness and perceived self-efficacy), and psychological distress and its 4 subfactors (somatic symptoms, anxiety/insomnia, social dysfunction, severe depression), for each group over time (T1, T2 and T3), we used a repeated measures ANOVA for the variables that followed a normal distribution and the non-parametric equivalent, the Friedman test, for the variables that did not follow a normal distribution. Effect sizes and 95% confidence intervals (95% CI) were calculated using Cohen’s d for the data from participants who completed the questionnaire at baseline and at follow-up. A value of 0.2 is generally interpreted as being suggestive of a small effect size, 0.5 of a medium effect size, and 0.8 of a large effect size [89].

A total of 63 students will be recruited for each group to detect 5% level of significance with 80% power in order to answer the research question (alpha: 0.05; Beta: 0.20 Power: 0.80; The variability (estimated) in (PSS / main variable): 2; Past experience (Feasibility Study), with similar experiments, with similar measurement method (PSS) and similar subjects, suggests that the data will be fairly distributed with an SD of 4; So, n = 2 × [(1.96 + 0.842) ^2^ × 4^2^] / 2^2^ = 62.809632).

The significance level was fixed at 0.05. All analyses were performed with SPSS (IBM v. 23) and Statistica (v. 12).

## Results

### Participants’ characteristics

The data in (Table 1) describe the study population, illustrating that the randomization protocol succeeded in balancing the intervention assignments and ensuring equal representation of important characteristics in the study’s population. There were no significant statistical differences between the two groups.

### Attrition

A total of 90 participants started the research study after being allocated into the two groups and sending an email confirming their participation after this stage, with 49 participants in the experimental group and 41 in the control group. A total of 55 students finished the study up to the first postassessment (of whom 20 were in the experimental group and 35 in the control group), and 47 students completed the second follow-up postassessment three months later (17 in the experimental group and 30 in the control group).

From the start of the intervention, in the experimental group (first session: 36 participants), there was a rate of general follow-up (for both groups) of 64.94% at the postassessment and 61.04% at the three-month follow-up (Table 2).

**Table 2:** Number of participants in each trial.

Trial	Experimental group				Control group
**Preintervention/*Recruitment* November 2015 to January 2016**	*64*				*64*
**Start/January 2016**	*49*				*41*
**Intervention**	**Session**				
**January to February 2016**	**1**^**st**^ *36*	**2**^**nd**^ *29*	**3**^**rd**^ *21*	**4th** *21*	
	Participants				
**Postintervention/March 2016**	*20*				*35*
**Follow-up/June 2016**	*17*				*30*

The attrition during the intervention went from 36 participants in the first session to 21 participants in the fourth session, with a follow-up rate of 58.33%.

### Preliminary analyses

Table 3 shows the means and SDs of the outcome variables at baseline and one-month and three-month follow-ups for the intervention and control groups.

**Table 3:** *Summary statistics for all outcome measures at baseline, postintervention and follow-up.

Variables	Preintervention								Post intervention M(SD)								Follow-up M(SD)							
	G1/n = 64				G2 /n = 64				G 1/n = 20				G2/n = 35				G1/n = 17				G2/n = 30			
	M (SD)	Md	Q1	Q3	M (SD)	Md	Q1	Q3	M (SD)	Md	Q1	Q3	M (SD)	Md	Q1	Q3	M (SD)	Md	Q1	Q3	M (SD)	Md	Q1	Q3
**Self esteem**	25.4 (5.7)	24	22	30	28.12 (5.9)	29.5	24	33	27.7 (5.9)	28	24	32	28.57 (5.6)	29	26	33	29 (3.1)	28	27	31.5	29.56 (6.02)	31	27.75	34.25
**Perceived stress**																								
Global	33.75 (6.3)	34	30	38	32.27 (5.7)	32.5	29	36	30.2 (7.2)	31	25	36	31.74 (6.5)	32	26	37	29.52 (5.6)	31	24.5	33.5	30.63 (7.09)	29.5	25.75	34.25
*Perceived helplessness*	22.06 (4.3)	22	19	25	21.1 (4.06)	21	18.25	24	19.5 (4.5)	19	16	23	20.71 (4.4)	20	16	24	19.17 (4.3)	19	16	21.5	19.86 (4.5)	18	17	24
*Perceived self-efficacy*	11.68 (2.5)	12	11	13	11.62 (2.3)	11	10	12.75	10.7 (3.2)	11	7.75	13	11.02 (2.4)	11	9	13	10.35 (2.1)	11	9	12.5	10.76 (3.09)	10.5	8	12.25
**Satisfaction in studies**	20.56 (6.6)	21	16	25	21.06 (5.5)	21	18	25.75	23.9 (8.2)	26	16	30	21.88 (7.1)	24	16	27	24 (7.2)	27	20	29	22.43 (7.6)	24	17.75	30
**General health**																								
Global	12.95 (4.2)	14	9	16	11.87 (3.8)	12	9	15	10.45 (3.7)	9.5	7	13	12.28 (3.1)	13	10	14	5.82 (4.1)	5	3	7	8.06 (6.7)	6	4	8.5
*Somatic symptoms*	3.35 (1.4)	3.5	2	4	3.01 (1.6)	3	2	4	2.1 (1.4)	1.5	1	3	3.82 (1.3)	4	3	5	2.05 (1.5)	2	1	3	2.4 (2.1)	2	1	4
*Anxiety and insomnia*	3.93 (2.4)	5	2	6	3.93 (2.1)	4	2	6	2.45 (2.7)	1.5	0	5.5	3.48 (2.07)	3	2	5	1.82 (2.3)	1	0	3.5	2.9 (2.1)	3	1	4
*Social dysfunction*	4.07 (1.7)	4	3	5.7	3.87 (1.7)	4	2	5.75	4.85 (1.5)	5	4	6	3.91 (1.9)	4	2	6	1.47 (1.6)	1	0	2	1.63 (1.8)	1	0	2.25
*Severe depression*	1.57 (1.9)	1	0	2	1.04 (1.7)	0	0	2	1 (1.4)	0.5	0	1	1.05 (1.7)	0	0	1	0.47 (0.87)	0	0	1	1.13 (2.33)	0	0	0.5

G1 = experimental group, G2 = control group. Italics = subfactor. M: mean. Md: median. Q1: first quartile. Q3: third quartile.

* Although we performed non-parametric tests, we chose to present the means and standard deviations in the tables for clarity of results. However, we also added the median and first and third quartiles as Summary statistics in the article.

Table 4 shows the comparisons between the two groups at each of the three stages. We noted only one significant difference at baseline between the two groups (U test: -2.49, p = 0.01): the self-esteem score (M = 28.12, SD = 5.9) was significantly higher in the control group than in the experimental group (M = 25.4, SD = 5.7).

**Table 4:** Comparison between both groups (Mann-Whitney test) in three trials.

Variables / questions	Preintervention		Postintervention		Follow-up	
	Z	*P*	Z	*P*	Z	*P*
**Self esteem**	-2.49	0.012*	-1.32	0.18	-0.88	0.37
**Perceived stress**						
*Global*	1.49	0.13	-0.6	0.47	-0.24	0.8
*Perceived helplessness*	1.28	0.19	-0.56	0.57	-0.19	0.84
*Perceived self-efficacy*	1.53	0.12	-0.23	0.81	-0.18	0.85
**Satisfaction in studies**	-0.39	0.69	1.62	0.1	0.66	0.5
**General health**						
*Global*	1.89	0.05	-2.16	0.03*	-1.18	0.23
*Somatic symptoms*	0.35	0.72	-3.63	0.001***	-0.25	0.79
*Anxiety and insomnia*	1.1	0.26	-2.78	0.04*	-1.81	0.06
*Social dysfunction*	-0.12	0.9	1.56	0.11	-0.12	0.9
*Severe depression*	1.72	0.08	0.6	0.54	0.01	0.99

Significant difference at

* p <.05;

** p <.01;

*** p <.001

At the postassessment stage, there were three significant differences between the two groups in global General Health (U test: -2.16, p = 0.03), with significantly lower scores in the experimental group (M = 10.45, SD = 3.7) than in the control group (M = 12.28, SD = 3.1). Regarding the two subfactors of the GHQ-28, the scores were significantly lower in the experimental group than in the control group for somatic symptoms (U test: -3.6 3, p = 0.001), with (M = 2.1, SD = 1.4) for the experimental group and (M = 3.82, SD = 1.3) for the control group, and for anxiety/insomnia (U test: -2.78, p = 0.04), with (M = 2.45, SD = 2.7) in the experimental group and (M = 3.48, SD = 2.07) in the control group.

The significant difference in self-esteem between the two groups at baseline disappeared at the postassessment and follow-up stages (Table 4)

### Intervention effects

Table 5 shows the means, standard deviations and effect sizes (Cohen’s d) of the variables at baseline and at one-month (or immediate) and three-month follow-ups in both the intervention and control groups.

**Table 5:** Means, standard deviations and effect sizes (Cohen’s d) of the observed and estimated marginal means for each trial.

	Mean (SD)			Effect sizes	
	Pre	Post	3-month follow-up	Pre to post	Pre to 3-month follow-up
**Experimental group = 17**					
**Self esteem**	24.3 (1.2)	28.1 (1.1)	29 (0.7)	3.2(2.2 to 4.3)	4.5 (3.3 to 5.8)
**Perceived stress**					
*Global*	34.3 (1.3)	29 (1.7)	29.5 (1.3)	-3.4 (-4.5 to -2.3)	-3.5 (-4.6 to -2.4)
*Perceived helplessness*	22.2 (1.08)	18.8 (1.1)	19.1 (1.05)	-3.1 (-4.1 to -2.1)	-2.9 (-3.8 to -1.9)
*Perceived self-efficacy*	12.05 (0.4)	10.1 (0.7)	10.3 (0.5)	-3.04 (-4.02 to -2.05)	-3.5 (-4.611 to -2.459)
**Satisfaction in studies**	21.2 (1.5)	25.7 (1.8)	24 (1.7)	2.6 (1.7 to 3.5)	1.6 (0.8 to 2.4)
**General health**					
*Global*	13.5 (1.08)	10.1 (0.8)	5.8(1.008)	-3.5 (-4.5 to -2.4)	-7.4 (-9.3 to -5.5)
*Somatic symptoms*	3.2 (0.3)	2 (0.3)	2.05 (0.3)	-3.7 (-4.9 to -2.6)	-3.3 (-4.4 to -2.3)
*Anxiety and insomnia*	4.4 (0.4)	2.1 (0.6)	1.8 (0.5)	-4.2 (-5.5 to -3.06)	-5.09 (-6.4 to -3.7)
*Social dysfunction*	4.1 (1.1)	5 (1.6)	1.4 (1.2)	0.5 (-0.1 to 1.2)	-2.2 (-3.06 to -1.3)
*Severe depression*	1.7 (2.1)	1.05 (1.5)	0.4 (0.8)	-0.3 (-1.06 to 0.2)	-0.7 (-1.4 to -0.09)
**Control group n = 30**					
**Self esteem**	29.6 (1.06)	29.5 (0.9)	29.5 (1.09)	-0.07 (-0.5 to 0.4)	-0.03 (-0.5 to 0.4)
**Perceived stress**					
*Global*	32.06 (1.1)	30.7 (1.1)	30.6 (1.2)	-1.09 (-1.6 to -0.5)	-1.1 (-1.6 to -0.6)
*Perceived helplessness*	20.6 (0.7)	20 (0.8)	19.8 (0.8)	-0.7 (-1.3 to -0.2)	-0.9 (-1.4 to -0.4)
*Perceived self-efficacy*	11.4 (0.5)	10.7 (0.4)	10.7 (0.5)	-1.3 (-1.9 to -0.8)	-1.2 (-1.8 to -0.6)
**Satisfaction in studies**	22.06 (1.1)	22.6 (1.2)	22.4 (1.3)	0.4 (-0.01 to 1.01)	0.2 (-0.2 to 0.7)
*General health*					
*Global*	11.6 (3.6)	12.03 (2.8)	8.06 (6.7)	0.1 (-0.3 to 0.6)	-0.6 (-1.1 to -0.1)
*Somatic symptoms*	3.03 (0.2)	3.8 (0.2)	2.4 (0.3)	3.006 (2.2 to 3.7)	-1.8 (-2.4 to -1.2)
*Anxiety and insomnia*	3.7 (0.4)	3.3 (0.3)	2.9 (0.3)	-1.064 (-1.6 to -0.5)	-2.1 (-2.7 to -1.4)
*Social dysfunction*	4.03 (1.8)	4.03 (1.9)	1.6 (1.8)	0 (-0.5 to—0.5)	-1.3 (-1.8 to -0.7)
*Severe depression*	0.8 (1.8)	0.8 (1.5)	1.1 (2.3)	0 (-0.5 to 0.5)	0.143 (-0.3 to 0.6)

ANOVA (repeated-measures or Friedman) (Table 6) revealed significant effects of the intervention over time for self-esteem (F2,32 = 7.23, p = 0.02); perceived stress (F2,32 = 5.28, p = 0.006) and its two subfactors “perceived helplessness” (F2,32 = 4.51, p = 0.1) and “perceived self-efficacy” (F2,32 = 5.31, p = 0.01); satisfaction in studies (F2,32 = 3.39, p = 0.02); and the global GHQ score (χ2 N = 17, dl = 2 = 15.11, p = 0.001) and three of its subfactors: somatic symptoms (F2,32 = 3.87, p = 0.03), anxiety/insomnia (F2,32 = 7.85, p = 0.001), and social dysfunction (χ2 N = 17, dl = 2 = 17.73, p = 0.001).

**Table 6:** ANOVA of repeated measures (F)/ Friedman test (χ2) intra-group.

Variable	ANOVA Repeated measures (F) Friedman test (χ2)*	*P*
**Experimental group n = 17**	***F = 2*.*32/*** *χ2:*N = 17*, *dl = 2*	
**Self esteem**	7.23	**0.002**
**Perceived stress**		
*Global*	5.82	**0.006**
*Perceived helplessness*	4.51	**0.01**
*Perceived self-efficacy*	5.31	**0.01**
**Satisfaction in studies**	3.93	**0.02**
**General health**		
*Global*	15.11	**0.001**
*Somatic symptoms*	3.87	**0.03**
*Anxiety and insomnia*	7.85	**0.001**
*Social dysfunction*	17.73	0.001*
*Severe depression*	5.09	0.07*
**Control group n = 30**	***F = 2*.*58/*** *χ2:*N = 30*, *dl = 2*	
**Self esteem**	1.18	**0.03**
**Perceived stress**		
*Global*	1.13	0.32
*Perceived helplessness*	0.74	0.47
*Perceived self-efficacy*	1.16	0.31
**Satisfaction in studies**	0.07	0.92
**General health**		
*Global*	15.2	0.001*
*Somatic symptoms*	7.25	**0.001**
*Anxiety and insomnia*	2.02	0.14
*Social dysfunction*	18.01	0.001*
*Severe depression*	0.92	0.62*

* = Friedman ANOVA

However, in the control group, we found significant changes over time regarding self-esteem (F2,58 = 1.18, p = 0.03); and the global GHQ score (χ2 N = 30, dl = 2 = 15.2, p = 0.001) and two of its subfactors: somatic symptoms (F2,58 = 7.25, p = 0.001) and social dysfunction (χ2 N = 30, dl = 2 = 18.01, p = 0.001) (Table 6).

### Assessment of the intervention by the participants

To obtain the participants’ opinions on the program, we asked a series of open- and closed-format questions to those who completed all the sessions. Table 7 shows the participants’ answers.

**Table 7:** Assessment of the online program by the participants.

Sample questions	Participants who finished the intervention = 20 Replies in n (%) X = No reply				
	Not at all 1	Slightly 2	Moderately 3	Very 4	Extremely 5
**1-Have you learned how to deal with stress better in general?**	1 (5)	X	8(40)	9(45)	2(10)
**2-Did you enjoy taking part in the online program?**	1 (5)	1 (5)	5 (25)	30%6(30)	7(35)
**3- Did you find the trials interesting?**	1 (5)	X	3 (15)	12(60)	4(20)
**4-Did you like how the sessions were presented?**	1 (5)	1(5)	5 (25)	8(40)	5(25)
**5-Would you now be able to give advice to your friends on how to deal with stress better?**	X	X	4 (20)	12 (60)	4(20)
**6-Did you enjoy taking part in each of the sessions?**	1 (5)	3(15)	7 (35)	4 (20)	5(25)
**7-Have you heard from anyone that you appeared less stressed?**	8(40)	6(30)	2 (10)	2(10)	2 (10)
**8- Did you exercise during the week after the trials?**	2 (10)	3(15)	7(35)	6(30)	2(10)
**9-Did you do your weekly activities?**	X	3(15)	6(30)	8(40)	3(15)
**10-Would you recommend this program to another student?**	1(5)	2(10)	3(15)	7(35)	7(35)

X = No reply. % () = percentage (frequency)

### Feedback on the intervention

We received comments throughout the program: after each exercise, after each session, and at the end of the program. These comments varied between positive feedback describing the effect a session or an exercise had on a participant, feedback on the sessions of exercises themselves, and interesting suggestions on how to improve the intervention, for instance, requests for more concrete examples of certain exercises.

## Discussion and conclusions

Our work assessed the efficacy of an online stress management program for university students.

We noted an improvement in self-esteem just after the intervention in the group that followed the program, which was maintained after three months of follow-up. As a reminder, before the intervention, self-esteem was higher in the control group than in the experimental group who benefitted from the intervention. However, this difference disappeared after the intervention and after the follow-up assessment three months later.

The results show a significant reduction in the experimental group between the start of the intervention and just after it for the anxiety/insomnia and somatic symptoms of the GHQ-28. However, this difference did not maintain significance after three months of follow-up. (Tables 2 and 3).

The significant decline of psychological distress levels measured by the global GHQ-28 score mirrors the results of the “Funambule” program study [75]. Our results are also similar in terms of the decrease in the level of anxiety [49] and the lack of significant differences between the two groups at follow-up, as also observed in a study by Chiauzzi and his team [49].

The fact that the differences between the groups at follow-up were not maintained could be explained by the fact that the participants in the control group already knew that they would soon be able to benefit from the intervention, which could potentially create positive effects.

The ANOVA analysis of the experimental group showed significant effects of time for all the variables studied in our research. Those variables included self-esteem; perceived stress and its two dimensions, perceived helplessness and perceived self-efficacy; satisfaction in studies; and psychological distress and its four dimensions: somatic symptoms, anxiety/insomnia, social dysfunction and severe depression.

The effect of time for the control group was noted a reduction trend of psychological distress and two of its dimensions, somatic symptoms and social dysfunction. And, on the other hand this effect was also found regarding the level of self-esteem, which also declined between baseline and follow-up three months later (Tables 4 and 5).

**Fig 2:**
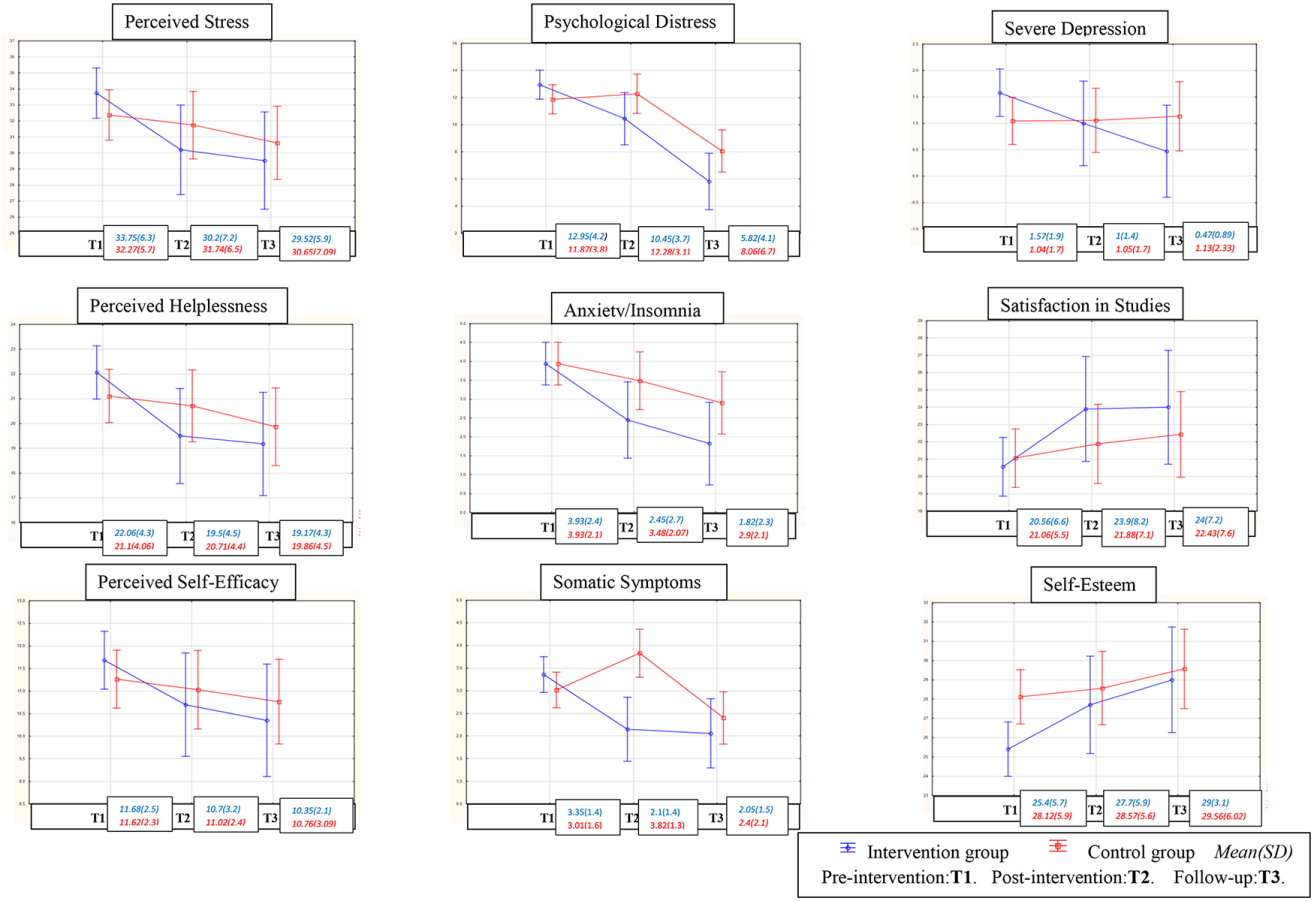
Mean scores over time based on the experimental condition for both groups and the studied mental health variables.

Despite the effect of time on both groups, we can note more effects in the group that received the intervention, as there were improvements for most of the studied variables in the experimental group but not the control group. The lack of maintenance of the effects at follow-up three months later in the experimental group (see Fig 2) is in agreement with a study carried out by Zetterqvist and his team [46].

**Intra-group effect sizes: for the experimental group**, large effect sizes (≥0.8) were observed for the measures of self-esteem, perceived stress, satisfaction in studies, and two dimensions of the GHQ-28: somatic symptoms and anxiety/insomnia. Moderate effect sizes (≥0.5) were noted for the social dysfunction dimension of the GHQ-28.

These moderate to large improvements were maintained after 3 months of follow-up. A smaller effect size (0.2) on another GHQ-28 subfactor, severe depression, was noted and maintained at follow-up. The effect sizes show an improvement from small to moderate after three months of follow-up.

**However, for the control group**, the effect sizes were small (0.2) for the variables of self-esteem, satisfaction in studies, and severe depression, and the effects sizes stayed small three months later. A small effect size for social dysfunction after the intervention increased to a moderate level three months later. For the variable of perceived stress and its two dimensions, somatic symptoms and anxiety/insomnia, the effect size was large and was maintained three months later.

We must highlight the fact that the comparison of effect sizes between the two groups showed a difference in favor of the experimental group, which mirrors the study by Zetterqvist and his team [46]. Regarding the variables mainly targeted by the program, for example, perceived stress, the effect sizes for the experimental group were (-3.443/-3.525) and for the control group, (-1.097 /-1.152). (Table 4).

These results are consistent with those of the study using the “Funambule” program [75], which found improvements in well-being and psychological distress such as anxiety.

These benefits from the intervention of an online stress management program on university students are in accordance with Zetterqvist and his team’s research [46], confirming the fact that an online intervention can have effects similar to those of a face-to-face intervention.

Our results are discordant with the study findings reported by Chiauzai et al. That study did not show any maintenance of significant differences in either group at a six-month follow-up [49]. However, some improvements were noted in this study [49] in terms of self-esteem and depression, in agreement with our results.

To conclude, the advantages of this type of Internet-based intervention relative to other programs lie in it being in a quite short and accessible format, which allows it to reach a larger number of students. Our results show significant effects immediately after the intervention on self-esteem and psychological distress (somatic symptoms and anxiety/insomnia) and an interesting improvement in all the studied variables, such as stress, depression and satisfaction in studies, at a follow-up three months later in the experimental group but not in the control group.

This online program could therefore be offered to students showing problems with stress who have not yet sought professional help. However, even though these results are similar to those obtained in the original program that inspired us, there are time constraints, as indicated by the absence of significant differences between groups three months after the intervention. To better determine the effects of time, a follow-up assessment at six months should be considered. The fact that the control group knows that they may benefit from the intervention should also be kept in mind.

## Limitations

Our work has a certain number of limitations: the size of the sample, the measures given by questionnaire only, a follow-up of only 3 months later, and the attacks of November 13th, 2015 in Paris, which could have had a psychological impact on the studied sample.

Moreover, over time there was a loss of participants; we faced this problem during the feasibility study in 2015, which led us to use an incentive, as Fridric and his team recommended [90]. Nevertheless, despite this incentive, our sample remained small. It is therefore still necessary to improve this intervention and pursue research that resolves the methodological problems raised by this type of intervention.

## Supporting information

S1 File CONSORT checklist. (DOC)

S2 File CONSORT. (DOC)

S3 File Trial protocol French version. (PDF)

S4 File Trial protocol English version. (DOCX)

S5 File The CNIL declaration no 1811031. (PDF)

## Abbreviations

ESDE: Satisfaction in Studies
GHQ-28: General Health Questionnaire
PSS-10: Perceived Stress Scale
RSES: Rosenberg Self-Esteem Scale
