# Supplementary material for: Can we learn to manage stress? A randomized controlled trial carried out on university students
Source: PLoS One. 2018 Sep 5;13(9):e0200997. doi: 10.1371/journal.pone.0200997 (PMC6124718; doi:10.1371/journal.pone.0200997)
Supplement: S2 File — (DOC) [file pone.0200997.s002.doc]

**
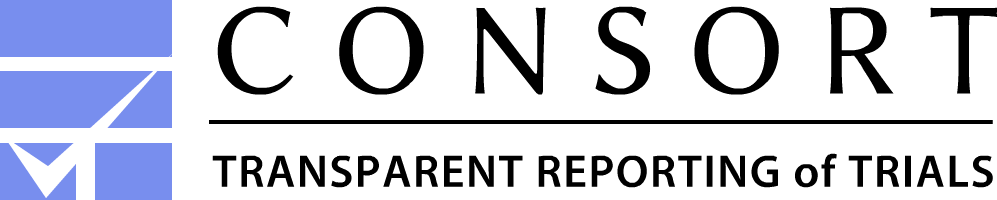
**

**Can we learn to manage stress? An experimental study carried out on university students**

**CONSORT 2010 Flow Diagram**

**Allocation**

**Post-intervention**

**Trial registration number (ISRCTN13709272)**

Assessed for eligibility (n= 142 )

Excluded (n= 14 )

♦  Not being aged between 18 and 30, (n= 14 )

After the intervention (n= 35 )

Control group

Allocated to intervention (n= 64 )

♦ Did not receive allocated intervention

The subjects of the control group were informed that they had been randomly put into the waiting group, that they would not be following the program during the study, and that they could follow it afterwards if they so wished.

After the intervention (n= 20)

Experimental group

Allocated to intervention (n=64 )

♦ Received allocated intervention

Randomized (n=128 )

After three month of the intervention (n= 17 )

After three month of the intervention (n= 30 )
