# Supplementary material for: Can we learn to manage stress? A randomized controlled trial carried out on university students
Source: PLoS One. 2018 Sep 5;13(9):e0200997. doi: 10.1371/journal.pone.0200997 (PMC6124718; doi:10.1371/journal.pone.0200997)
Supplement: S3 File — (PDF) [file pone.0200997.s003.pdf]

Dalia Saleh   Mai   2015

Etude sur une intervention de gestion du stress par internet pour les étudiants universitaires en France

Dalia Saleh, Nathalie Camart, Lucia Romo.

Madame, Mademoiselle, Monsieur,

Dans le cadre d'une étude en cours à l'Université de Paris Ouest Nanterre La Défense, nous présentons un projet-pilote de gestion du stress par Internet qui s'inspire du programme Funambule : Pour une gestion équilibrée du stress. Il s'agit d'une version proposée dans un contexte différent (Internet) du programme Funambule. Ce projet-pilote a été développé et adapté dans l'objectif de proposer une intervention de gestion du stress à des étudiants à l'université. Elle vise à aider les étudiants à mieux gérer leur stress par la passation de quatre séances, qui vous seront proposées dans les parties suivantes.

Introduction

Les étudiants à l'université constituent une catégorie de personnes particulièrement vulnérables au stress (Robotham & Julian, 2006) et qui présente selon la littérature une niveau élevé de celui-ci (Guille, & Sen, 2013; Montero-Marín, Demarzo, Stapinski, Gili, & García-Campayo, 2014; Vandentorren, Verret, Vignonde, & Maurice-Tison, 2005). Ils sont susceptibles également de présenter des problèmes liés au stress comme l'anxiété, la dépression (Feeney, Hussey, & Donnellan, 2010), les troubles alimentaires (Lavender, De Young, & Anderson, 2010; Luce, Crowther, & Pole, 2008), les problème de sommeil (Pilcher, Ginter, & Sadowsky, 1997). Ces taux sont supérieurs au niveau de morbidité psychologique déclaré en population générale (Adlaf, Gliksman, Demers, & Newton-Taylor, 2001; Baykan, Naçar, & Cetinkaya, 2012; Bewick, Koutsopoulou, Miles, Slaa, & Barkham, 2010; Mor; O'Brien, Mathieson, Leafman, & Rice-Spearman, 2012).

En fait, la prévalence de plusieurs troubles psychiques est toujours importante chez les étudiants universitaires quelles que soient les études. Par exemple, le taux de détresse psychique varie entre 21 % et 82% (Dyrbye et al., 2011; Humphris et al., 2002; Nerdrum, Rustøen, & Rønnestad, 2006; Strenna, Chahraoui, & Vinay, 2009), le taux de la dépression entre 53 %, et 13% (Boujut, Koleck, Bruchon-Schweitzer, & Bourgeois, 2009; Dahlin, Joneborg, & Runeson, 2005; Furr, Westefeld, McConnell, & Jenkins, 2001; Wong, Cheung, Chan, Ma, & Tang, 2006), l'anxiété entre 47% et 34% (Bayram & Bilgel, 2008; Shamsuddin et al., 2013; Wong et al., 2006) et le taux du stresse varie entre 79% et 33 % (Bughi, Sumcad,

Université de Paris Ouest Nanterre la Défense
EA 4430 - CLIPSYD
Psychologie Clinique,
Psychanalyse,
Développement
200 Avenue de la République, 92000 Nanterre

& Bughi, 2006; O'Brien et al., 2012; Vandentorren et al., 2005). 50% des étudiants qui ont déclaré avoir des problèmes de santé mentale comme la dépression, l'anxiété et des pensées suicidaires, n'ont pas cherché de l'aide (Zivin, Eisenberg, Gollust, & Golberstein, 2009).

Internet est devenu un outil essentiel dans le champ des interventions de type « self-help ». Il peut se définir comme le fait de permettre un accès en ligne à un programme à visée thérapeutique. Ce type d'intervention en self-help via internet présente plusieurs objectifs comme la réduction des risques concernant des problèmes visés tels que le niveau de stress, d'anxiété et de dépression (Zetterqvist, Maanmies, Ström, & Andersson, 2003), comme augmenter le niveau de bien-être (Vliet & Andrews, 2009), améliorer les stratégies d'adaptation (George, Dellasega, Whitehead, & Bordon, 2013), et même augmenter l'activité physique hebdomadaire (Chiauzzi, Brevard, Thurn, Decembrele, & Lord, 2008). Les avantages de ces interventions en ligne seraient leur accessibilité et disponibilité avec accès possible à tout moment, la confidentialité et la discrétion surtout pour les individus qui ne souhaitent pas consulter dans un centre de soins, et la possibilité de diffuser le programme à de larges populations concernées et de façon économique.

L'objectif de cette recherche est de mesurer l'efficacité d'une intervention de gestion du stress via internet auprès d'étudiants universitaires sur des variables de santé psychiques : l'estime de soi, le stress perçu et ses deux sous-facteurs (le débordement perçu, l'efficacité personnelle perçue), la détresse psychologique et ses quatre sous-facteurs (symptômes somatiques, anxiété/insomnie, dysfonctionnement social, dépression sévère) ainsi que la satisfaction dans les études.

Matériel et méthode

Ce protocole propose un essai contrôlé randomisé pour déterminer l'efficacité d'une intervention de gestion du stress via internet auprès d'étudiants universitaires.

Les critères d'inclusion sont : être étudiant/e dans une université française ; maitriser le français ; âge entre 18 et 30 ; avoir un adresse mail et accès à l'Internet.

Le protocole de cette recherche propose une essaie contrôlée randomisée pour déterminer l'efficacité d'une intervention de gestion du stress par internet.

Les sujets vont été répartis aléatoirement d'un l'une de deux groupes (expérimentale ou contrôle). Les participants vont été évalué en trois temps (pré-évaluation, post-

Université de Paris Ouest Nanterre la Défense
EA 4430 - CLIPSYD
Psychologie Clinique,
Psychanalyse,
Développement
Avenue de la République, 92000 Nanterre

évaluation/directement après l'intervention, en follow-up/après trois mois de suivi, et après six mois de suivi)

Il faut noter que toutes les informations concernant les participants seront traitées de façon confidentielle et anonyme.

Le e-mail des participants n'apparaîtra dans aucun document ni enregistrement en rapport avec cette recherche. Les données recueillies feront l'objet d'un traitement statistique collectif respectant l'anonymat.

Les participants seront informés que nous demandons de répondre aux exercices de façon spontanée. Il n'y a pas de bonnes ni de mauvaises réponses.

Plus précisément, le protocole pour chaque groupe :

Concernant le groupe expérimental :

- Participer à cette recherche implique de suivre les quatre séances : une séance de 20 minutes chaque semaine (comprenant tous les exercices), de réaliser les tâches à domicile entre les séances et de remplir l'évaluation après l'intervention.
- Dès que nous allons recevoir votre réponse pour la première évaluation, le nom de participant entrera dans la liste des participants pour le tirage au sort pour les chèques cadeaux de 20 euros.

Concernant le groupe contrôle :

- Les participants dans le groupe contrôle seront informés que leur noms a été choisi pour participer à la deuxième application de l'intervention.
- Ils seront informés qu'il faut répondre à la batterie de questionnaires (pré-évaluation) , que ils ont déjà rempli pour que leur nom soit dans liste des participants pour la tombola afin de gagner un chèque cadeau de 20 euros (au mois de mars 2016).

Incitation :

L'étude permet de participer à un tirage au sort d'un chèque cadeau

Université de Paris Ouest Nanterre la Défense
EA 4430 - CLIPSYD
Psychologie Clinique, Psychanalyse, Développement
200, Avenue de la République, 92000 Nanterre

- Il faut noter que le participant sera libre de participer ou non à cette recherche et que s'il accepte, il est libre de s'en retirer à tout moment sans aucune conséquence (mai il ne participera pas au tirage au sort du chèque cadeau).

- Les participants seront informés qu'ils ont une semaine pour répondre à la batterie de questionnaires. Passée une semaine, ils peuvent continuer la participation, mais, ils perdent le droit de recevoir le chèque cadeau.

- Le tirage au sort aura lieu à l'université de Nanterre, les participants pourront y assister, sinon, ils doivent laisser les coordonnées pour qu'on puisse envoyer la date et le lieu de la remise des chèques.

Participants

L'échantillon sera composé d'étudiants universitaires en France. Il y aura deux groupes, « groupe expérimental» et « groupe contrôle » :

En janvier, les étudiants dans le groupe d'intervention seront contactés pour donner leur accord pour participer à cette intervention et effectuer « les exercices » entre les séances. Ils vont être invités à visiter le site web une fois par semaine et à y passer au moins 20 minutes. Ils auront la possibilité de se connecter plus longtemps ou plus fréquemment s'ils le souhaitent. Au même moment, les sujets dans le groupe contrôle vont être informés qu'ils seront répartis aléatoirement dans le groupe d'attente et qu'ils pourront suivre le programme plus tard s'ils le souhaitent.

Recrutement et Procédure

La recherche sera présentée comme une étude portant sur l'évaluation d'une intervention de gestion du stress pour des étudiants à l'université, dont la participation serait anonyme et volontaire.

Le recrutement sera lancé principalement au sein du site de l'université Paris Nanterre, qui va mettre en ligne l'annonce de la recherche, ce qui va permettre la diffusion dans newsletter des étudiants et sur les réseaux sociaux de l'université. Des affiches seront également diffusés dans le campus et via des réseaux sociaux.

Université de Paris Ouest Nanterre la Défense
EA 4430 - CLIPSYD
Psychologie Clinique, Psychanalyse, Développement
200, Avenue de la République, 92000 Nanterre

Instruments d'évaluation :

Les participants vont répondre à une batterie de questionnaires contenant quatre questionnaires mis en ligne : en " pré " (avant l'intervention) et en " post " évaluation (après l'intervention). Ces questionnaires sont les suivants :

Une échelle de stress perçu «Perceived Stress Scale» [PSS 10]. Il comporte deux notes seuils : un score-seuil supérieur à 24 pour la dimension d'anxiété et supérieur à 26 pour la dimension de dépression (Collange, Bellinghausen, Chappé, Saunder, & Albert, 2013)

L'échelle d'estime de soi de Rosenberg « Rosenberg's Self-Esteem Scale » [RSE]: une note inférieure à 30 indique une faible estime de soi et une note supérieure ou égale à 30 indique une bonne estime de soi (Vallieres & Vallerand, 1990 ; Chabrol et al., 2004)

Une échelle de satisfaction de vie dans les études «Scale of Satisfaction in Studies» [ESDE] validée en français par Vallerand et Bissonette (1990) (Vallerand & Bissonnette, 1990)

Un questionnaire de détresse psychologique « General Health Questionnaire » [GHQ-28] : une note égale ou supérieure à 5 indique une détresse psychologique (Goldberg & Hillier, 1979) (Bolognini, Bettschart, Zehnder-Gubler, & Rossier, 1989)

Les étudiants auront également rempli un auto-questionnaire comprenant des questions sociodémographiques (âge, sexe, année d'étude, filière…).

Intervention de gestion du stress par internet « Je gère mon stress »

Dans la seconde étape de ce travail, nous avons établi un projet-pilote de gestion du stress par internet qui s'inspire du programme « Funambule » développé par Dumont et son équipe (2012) au Canada(Dumont, 2012). Des techniques de TCC, que nous avons adapté sur internet, sont utilisées dans ce programme. Une séance d'évaluation avant et après le module sera proposée. Un email va être crée pour contacter les étudiants si celui-ci n'a pas visité le site internet, et également pour nous contacter dans le cas de questions éventuelles sur la gestion du stress ou concernant des problèmes techniques dans l'utilisation du programme. Les étudiants seront informés puis ont donné leur accord pour participer à cette intervention et

Université de Paris Ouest Nanterre la Défense
EA 4430 - CLIPSYD
Psychologie Clinique,
Psychanalyse,
Développement
200, Avenue de la République, 92000 Nanterre

effectuer les tâches entre les séances. Ils seront invités à visiter le site web une fois par semaine et à y passer au moins 30 minutes. Néanmoins, ils sont libres de se connecter plus longtemps que l'intervention (en moyenne de 20 minutes par semaine) ou plus fréquemment s'ils le souhaitent. Ce programme vise à aider les étudiants à apprendre des techniques faciles et efficaces pour mieux faire face aux situations stressantes. L'intervention se compose de quatre séances de 20 minutes chacune comprenant des exercices pratiques et une à deux activités hebdomadaires que le participant est invité à accomplir.

Thèmes de l'intervention de gestion du stress via internet :

La définition du stress : Tolérer le changement ; Accepter un certain degré du stress ; Comprendre le stress ; Regarder toujours la moitié pleine du verre ; Mesurer le niveau de stress ; Déterminer les sources de stress ; Lister les peurs ; Conjuguer sa vie au présent.

Exercices sur le corps : Mes symptômes de stress ; Prendre conscience de ses tensions ; La respiration en ballon ; La méthode de relaxation " contracte- relâche " ; Créer ses propres "mantras" de détente ; Visualiser un endroit calme et sécurisant.

Exercices sur les pensées : Le pouvoir de ma pensée ; Les pensées stressantes qui nuisent à ma concentration ; La tâche des cinq colonnes de Beck ; Remplacer une pensée non aidante par une pensée aidante ; Se méfier de des interprétations hâtives non fondées ; Méthode d'auto-instruction de Meichenbaum ; La démarche de résolution de problème.

Exercices sur les stratégies adaptatives : Ajuster sa stratégie à son pouvoir d'action ; Rappeler et vérifier ; Améliorer sa gestion du temps ; Se préparer à un examen ; Vérifier son niveau de stress ; Utiliser les cartes antistress ; Conseiller une personne stressée.

Références :

Adlaf, E. M., Gliksman, L., Demers, A., & Newton-Taylor, B. (2001). The Prevalence of Elevated Psychological Distress Among Canadian Undergraduates: Findings from the 1998 Canadian Campus Survey. *Journal of American College Health, 50*(2), 67–72. https://doi.org/10.1080/07448480109596009

Baykan, Z., Naçar, M., & Cetinkaya, F. (2012). Depression, anxiety, and stress among last-year students at Erciyes University Medical School. *Academic Psychiatry: The Journal Of The American Association Of Directors Of Psychiatric Residency Training*,

Université de Paris Ouest Nanterre la Défense EA 4430 - CLIPSYD Psychologie Clinique, Psychanalyse, Développement [illegible] République, 92000 Nanterre

And The Association For Academic Psychiatry, *36*(1), 64–65. https://doi.org/10.1176/appi.ap.11060125

Bayram, N., & Bilgel, N. (2008). The prevalence and socio-demographic correlations of depression, anxiety and stress among a group of university students. *Social Psychiatry and Psychiatric Epidemiology*, *43*(8), 667–672. https://doi.org/10.1007/s00127-008-0345-x

Bewick, B., Koutsopoulou, G., Miles, J., Slaa, E., & Barkham, M. (2010). Changes in undergraduate students' psychological well-being as they progress through university. *Studies in Higher Education*, *35*(6), 633–645. https://doi.org/10.1080/03075070903216643

Bolognini, M., Bettschart, W., Zehnder-Gubler, M., & Rossier, L. (1989). The validity of the French version of the GHQ-28 and PSYDIS in a community sample of 20 year olds in Switzerland. *European Archives of Psychiatry and Neurological Sciences*, *238*(3), 161–168. https://doi.org/10.1007/BF00451005

Boujut, E., Koleck, M., Bruchon-Schweitzer, M., & Bourgeois, M. L. (2009). Mental health among students: A study among a cohort of freshmen. *Annales Médico-Psychologiques*, *167*(9), 662–668. https://doi.org/10.1016/j.amp.2008.05.020

Bughi, S. A., Sumcad, J., & Bughi, S. (2006). Effect of brief behavioral intervention program in managing stress in medical students from two southern California universities. *Medical Education Online*, *11*, 1–8.

Chabrol, H., Carlin, E., Michaud, C., Rey, A., Cassan, D., Juillot, M., … Callahan, S. (2004). Étude de l'échelle d'estime de soi de Rosenberg dans un échantillon de lycéens. *Neuropsychiatrie de l'Enfance et de l'Adolescence*, *52*(8), 533–536. https://doi.org/10.1016/j.neurenf.2004.09.007

Chiauzzi, E., Brevard, J., Thurn, C., Decembrele, S., & Lord, S. (2008). MyStudentBody–Stress: An online stress management intervention for college students. *Journal of Health Communication*, *13*(6), 555–572. https://doi.org/10.1080/10810730802281668

Collange, J., Bellinghausen, L., Chappé, J., Saunder, L., & Albert, E. (2013). Stress perçu : à partir de quel seuil devient-il un facteur de risque pour les troubles anxiodépressifs ? *Archives Des Maladies Professionnelles et de l'Environnement*, *74*(1), 7–15. https://doi.org/10.1016/j.admp.2012.12.009

university. *BMC Psychiatry*, *15*, 26. https://doi.org/10.1186/s12888-015-0403-3

Université de Paris Ouest Nanterre la Défense
EA 4430 - CLIPSYD
Psychologie Clinique, Psychanalyse, Développement

Dahlin, M., Joneborg, N., & Runeson, B. (2005). Stress and depression among medical students: a cross-sectional study. *Medical Education*, *39*(6), 594–604. https://doi.org/10.1111/j.1365-2929.2005.02176.x

Deasy, C., Coughlan, B., Pironom, J., Jourdan, D., & Mannix-McNamara, P. (2014). Psychological Distress and Coping amongst Higher Education Students: A Mixed Method Enquiry. *PLOS ONE*, *9*(12), e115193. https://doi.org/10.1371/journal.pone.0115193

Dumont, M. (2012). *Funambule Pour une gestion équilibrée du stress*. Québec: Septembre éditeur. Retrieved from http://www.septembre.com/livres/funambule-1259.html

Dyrbye, L. N., Harper, W., Durning, S. J., Moutier, C., Thomas, M. R., Massie, F. S., Jr., … Shanafelt, T. D. (2011). Patterns of distress in US medical students. *Medical Teacher*, *33*(10), 834–839. https://doi.org/10.3109/0142159x.2010.531158

Furr, S. R., Westefeld, J. S., McConnell, G. N., & Jenkins, J. M. (2001). Suicide and depression among college students: A decade later. *Professional Psychology: Research and Practice*, *32*(1), 97. https://doi.org/10.1037/0735-7028.32.1.97

George, D. R., Dellasega, C., Whitehead, M. M., & Bordon, A. (2013). Facebook-based stress management resources for first-year medical students: A multi-method evaluation. *Computers in Human Behavior*, *29*(3), 559–562. https://doi.org/10.1016/j.chb.2012.12.008

Goldberg, D. P., & Hillier, V. (1979). A scaled version of the General Health Questionnaire. *Psychological Medicine*, *9*(1), 139–145.

Grant, F., Guille, C., & Sen, S. (2013). Well-Being and the Risk of Depression under Stress. *PLOS ONE*, *8*(7), e67395. https://doi.org/10.1371/journal.pone.0067395

Humphris, G., Blinkhorn, A., Freeman, R., Gorter, R., Hoad-Reddick, G., Murtomaa, H., … Splieth, C. (2002). Psychological stress in undergraduate dental students: baseline results from seven European dental schools. *European Journal of Dental Education*, *6*(1), 22–29. https://doi.org/10.1034/j.1600-0579.2002.060105

Koochaki, G. M., Charkazi, A., Hasanzadeh, A., Saedani, M., Qorbani, M., & Marjani, A. (2011). Prevalence of stress among Iranian medical students: a questionnaire survey. *Eastern Mediterranean Health Journal*, *17*(7), 593–598.

Lavender, J. M., De Young, K. P., & Anderson, D. A. (2010). Eating Disorder Examination Questionnaire (EDE-Q): Norms for undergraduate men. *Eating Behaviors*, *11*(2), 119–121. https://doi.org/10.1016/j.eatbeh.2009.09.005

Université Paris Ouest Nanterre la Défense
EA 4430 - CLIPSYD
Psychologie Clinique,
Psychanalyse,
Développement
200, Avenue de la République, 92000 Nanterre

Luce, K. H., Crowther, J. H., & Pole, M. (2008). Eating Disorder Examination Questionnaire (EDE-Q): Norms for undergraduate women. *International Journal of Eating Disorders*, *41*(3), 273–276. https://doi.org/10.1002/eat.20504

Montero-Marín, J., Demarzo, M. M. P., Stapinski, L., Gili, M., & García-Campayo, J. (2014). Perceived Stress Latent Factors and the Burnout Subtypes: A Structural Model in Dental Students. *PLOS ONE*, *9*(6), e99765. https://doi.org/10.1371/journal.pone.0099765

Nerdrum, P., Rustøen, T., & Rønnestad, M. H. (2006). Student Psychological Distress: A psychometric study of 1750 Norwegian 1st-year undergraduate students. *Scandinavian Journal of Educational Research*, *50*(1), 95–109. https://doi.org/10.1080/00313830500372075

O'Brien, L., Mathieson, K., Leafman, J., & Rice-Spearman, L. (2012). Level of stress and common coping strategies among physician assistant students. *The Journal Of Physician Assistant Education: The Official Journal Of The Physician Assistant Education Association*, *23*(4), 25–29. https://doi.org/10.1097/01367895-201223040-00006

Pilcher, J. J., Ginter, D. R., & Sadowsky, B. (1997). Sleep quality versus sleep quantity: Relationships between sleep and measures of health, well-being and sleepiness in college students. *Journal of Psychosomatic Research*, *42*(6), 583–596. https://doi.org/10.1016/S0022-3999(97)00004-4

Robotham, D., & Julian, C. (2006). Stress and the higher education student: a critical review of the literature. *Journal of Further and Higher Education*, *30*(2), 107–117. https://doi.org/10.1080/03098770600617513

Schulz, K. F., Altman, D. G., & Moher, D. (2010). CONSORT 2010 Statement: updated guidelines for reporting parallel group randomised trials. *BMJ*, *340*, c332. https://doi.org/10.1136/bmj.c332

Shamsuddin, K., Fadzil, F., Ismail, W. S. W., Shah, S. A., Omar, K., Muhammad, N. A., … Mahadevan, R. (2013). Correlates of depression, anxiety and stress among Malaysian university students. *Asian Journal Of Psychiatry*, *6*(4), 318–323. https://doi.org/10.1016/j.ajp.2013.01.014

Stallman, H. M. (2010). Psychological distress in university students: A comparison with general population data. *Australian Psychologist*, *45*(4), 249–257. https://doi.org/10.1080/00050067.2010.482109

Université Paris Ouest Nanterre la Défense
EA 4430 - CLIPSYD
Psychologie Clinique,
Psychanalyse,
Développement
200, Avenue de la République, 92000 Nanterre

Strenna, L., Chahraoui, K., & Vinay, A. (2009). Santé psychique chez les étudiants de première année d'école supérieure de commerce: liens avec le stress de l'orientation professionnelle, l'estime de soi et le coping. *L'orientation scolaire et professionnelle*, (38/2), 183–204. https://doi.org/10.4000/osp.1902

Vallerand, R. J., & Bissonnette, R. (1990). Construction et validation de l'Échelle de Satisfaction dans les Études (ESDE). [Construction and validation of the Scale of Satisfaction in Studies.]. *Canadian Journal of Behavioural Science/Revue Canadienne Des Sciences Du Comportement*, *22*(3), 295–306. https://doi.org/10.1037/h0078987

Vallieres, E. F., & Vallerand, R. J. (1990). Traduction et validation canadienne-française de l'échelle de l'estime de soi de Rosenberg. *International Journal of Psychology*, *25*(2), 305–316. https://doi.org/10.1080/00207599008247865

Vandentorren, S., Verret, C., Vignonde, M., & Maurice-Tison, S. (2005). Besoins d'information en santé des étudiants au service inter-universitaire de médecine préventive de Bordeaux. *Santé publique*, *17*(1), 47–56. https://doi.org/10.3917/spub.051.0047

Vliet, H. V., & Andrews, G. (2009). Internet-based course for the management of stress for junior high schools. *Australasian Psychiatry*, *43*(4), 305–309. https://doi.org/10.1080/00048670902721145

Walsh, J. M., Feeney, C., Hussey, J., & Donnellan, C. (2010). Sources of stress and psychological morbidity among undergraduate physiotherapy students. *Physiotherapy*, *96*(3), 206–212. https://doi.org/10.1016/j.physio.2010.01.005

Wong, J. G. W. S., Cheung, E. P. T., Chan, K. K. C., Ma, K. K. M., & Tang, S. W. (2006). Web-based survey of depression, anxiety and stress in first-year tertiary education students in Hong Kong. *The Australian And New Zealand Journal Of Psychiatry*, *40*(9), 777–782. https://doi.org/10.1111/j.1440-1614.2006.01883.x

Zetterqvist, K., Maanmies, J., Ström, L., & Andersson, G. (2003). Randomized controlled trial of Internet-based stress management. *Cognitive Behaviour Therapy*, *32*(3), 151–160. https://doi.org/10.1080/16506070310016248

Zivin, K., Eisenberg, D., Gollust, S. E., & Golberstein, E. (2009). Persistence of mental health problems and needs in a college student population. *Journal of Affective Disorders*, *117*(3), 180–185. https://doi.org/10.1016/j.jad.2009.01.001

Université de Paris Ouest Nanterre la Défense
EA 4430 - CLIPSYD
Psychologie Clinique,
Psychanalyse,
Développement
200, Avenue de la République, 92000 Nanterre
