# Supplementary material for: Can we learn to manage stress? A randomized controlled trial carried out on university students
Source: PLoS One. 2018 Sep 5;13(9):e0200997. doi: 10.1371/journal.pone.0200997 (PMC6124718; doi:10.1371/journal.pone.0200997)
Supplement: S4 File — (DOCX) [file pone.0200997.s004.docx]

**Can we learn to manage stress? A Randomized Controlled trial carried out on university students**

Dalia Saleh, Nathalie Camart, Lucia Romo

As part of an ongoing study at Paris Nanterre University, we present a pilot project to manage stress online, inspired by an existing program “Funambule: For a web-based management of stress”. This is a version proposed in a different context from the Funambule program, since it will be done online. This pilot project was developed and adapted with the objective to propose a stress management intervention to university students, and aims to help students to better manage their stress in four sessions.

# Introduction

University students make up a group of people who are particularly vulnerable to stress (Robotham & Julian, 2006) and present, according to scientific literature, a high level of it (Guille, & Sen, 2013; Montero-Marín, Demarzo, Stapinski, Gili, & García-Campayo, 2014; Vandentorren, Verret, Vignonde, & Maurice-Tison, 2005). They are prone to have stress-related issues such as anxiety, depression (Walsh, Feeney, Hussey, & Donnellan, 2010), eating disorders (Lavender, De Young, & Anderson, 2010; Luce, Crowther, & Pole, 2008),, and insomnia (Pilcher, Ginter, & Sadowsky, 1997). These rates are higher in terms of psychological morbidity than those declared in a general population (Adlaf, Gliksman, Demers, & Newton-Taylor, 2001; Baykan, Naçar, & Cetinkaya, 2012; Bewick, Koutsopoulou, Miles, Slaa, & Barkham, 2010; Mor; O’Brien, Mathieson, Leafman, & Rice-Spearman, 2012)..

In fact, several mental health issues in this group has always been prevalent, regardless of the level of studies. For example, mental health distress rate is between 21 % and 82% (Dyrbye et al., 2011; Humphris et al., 2002; Nerdrum, Rustøen, & Rønnestad, 2006; Strenna, Chahraoui, & Vinay, 2009), the rate of depression between 53 %, and 13(Boujut, Koleck, Bruchon-Schweitzer, & Bourgeois, 2009; Dahlin, Joneborg, & Runeson, 2005; Furr, Westefeld, McConnell, & Jenkins, 2001; Wong, Cheung, Chan, Ma, & Tang, 2006), anxiety between 47% and 34% (Bayram & Bilgel, 2008; Shamsuddin et al., 2013; Wong et al., 2006) and the rate of stress varies between 79% and 33 % (Bughi, Sumcad, & Bughi, 2006; Koochaki et al., 2011; O’Brien et al., 2012; Vandentorren et al., 2005). 50% of students who claimed to suffer from mental health issues such as depression, anxiety and suicidal thoughts, did not seek help (Zivin, Eisenberg, Gollust, & Golberstein, 2009).

The Internet has become an essential tool in the field of “self-help” interventions. It can be defined by the fact that it allows an online access to a therapeutically aimed program. This type of intervention via the Internet has several goals, such as reduction of risks leading to the targeted problem, the level of stress, anxiety and depression (Zetterqvist, Maanmies, Ström, & Andersson, 2003), the level of well-being (Vliet & Andrews, 2009), to enhance adaptation strategies (George, Dellasega, Whitehead, & Bordon, 2013), and even to increase the amount of weekly physical activities (Chiauzzi, Brevard, Thurn, Decembrele, & Lord, 2008). The advantages of these online trials would be their accessibility and availability, considering the possibility to access it at any time, their confidentiality, and their discretion especially for people who do not want to seek medical help in a health center, as well as the opportunity to spread the program to a large population, in an economical way.

The aim of this research is to measure the efficiency of an online stress management program on university students based on mental health variables: self-esteem, perceived stress and its two sub factors (feeling overloaded/overwhelmed and perceived personal efficiency), psychological distress and its four sub factors (somatic symptoms, anxiety/insomnia, social dysfunction, severe depression) as well as satisfaction in studies.

**Materials and Methods**

**Study design**

This protocol offers a randomized controlled trial to determine the efficiency of an online stress management intervention on university students.

The inclusion criteria are: being a student at a French university, having mastered the French language, being aged between 18 and 30, and having an e-mail address and access to the Internet.

The subjects have been randomly allocated into one of the two groups: the experimental group (those who followed the program) and the control group (those who did not follow the program).

They will be evaluated in three different periods (pre-evaluation, post-evaluation immediately after intervention, and in follow up after three month and after six month).

The information regarding the subjects will remain confidential and anonymous.

Their e-mail addresses will not show up in any document or records related to this research. The statistical data will be processed collectively and anonymously.

The subjects will be informed that we shall ask them to answer spontaneously to the exercises. There are no right or wrong answers.

**The protocol for each group will be described as the following:**

Concerning the experimental group:

- Participation in this research takes part in four sessions : a 20 minute session each week (including all the exercises), tasks to be completed at home between the sessions, and to complete an evaluation after the intervention
- As soon as we receive your answers for the first evaluation, the subject’s name will be included in the list of subjects for the lottery to win a gift voucher.

Concerning the control group:

- Subjects in the control group will be informed that their names have been chosen to take part in the second application intervention.
- They will be informed that they have to answer several questionnaires (pre-evaluation), so that they can rest assured that their names will be included in a list eligible for the lottery to win a gift voucher valued at 20 euros (in March).
- **Incitation**

**Contributing to the study will allow participation in a lottery for a gift voucher**

- the subject will be free to participate or not in this research and if they do, they will be free to retire at any moment without any consequences (but they won’t participate in the lottery).
- The subjects will be informed that they have to answer the questionnaires within the week. After the delay, they can still participate but will not be eligible for winning the gift voucher anymore.
- The lottery will take place at Nanterre University. The subjects will be allowed to stay in, either they can give their contact information so as they can be sent the moment and the place.

**Subjects**

The sample will be composed of French university students. There will be two groups, the experimental group and the control group.

In January, students that were allocated into the experimental group were informed of that. They then gave their consent to take part in the intervention and to perform tasks between sessions. They were invited to visit the website once a week and to spend at least 20 minutes on it. At the same time, the subjects in the control group were informed that they had been randomly allocated into the control group and that they could follow the program afterwards if they so wished.

**Recruitment and Procedure**

The research has been presented as a study on the assessment of a stress management intervention carried out on university students, whose participation was anonymous and voluntary.

The recruitment took place mostly on the Paris Nanterre University campus, and an advertisement for our research was published only by the university as well. This allowed publicity in the student newsletter and on the university’s social media networks. Posters were also put up in the campus and posted digitally on social media.

**Measures**

A battery of questionnaires, including four uploaded questionnaires during the four stages (pre-evaluation, post-evaluation, assessment and follow-up).

- Stress was assessed by the Perceived Stress Scale (PSS-10) using the 10-item concept (Bellinghausen, Collange, Botella, Emery, & Albert, 2009).
- To measure self-esteem we decided to use a 10-item version of the Rosenberg Self-Esteem Scale (RSES) (Chabrol et al., 2004; Vallieres & Vallerand, 1990).
- In order to evaluate the participants’ satisfaction in studies, we opted for the 5-item version of (ESDE) (Vallerand & Bissonnette, 1990).
- We measured psychological distress by using the 28-item General Health Questionnaire (GHQ-28) (Bolognini, Bettschart, Zehnder-Gubler, & Rossier, 1989; Goldberg & Hillier, 1979)

The students filled in socio-demographic data, such as their age, their gender, their age, where they have lived, their year of studies, their study program and university of origin.

**Intervention (« *I’m managing my stress »*) :**

We created an online stress management pilot program titled *I’m managing my stress,*inspired by the *Funambule* program developed by Dumont and his team (40) in Canada, in which cognitive-behavioral therapy techniques were used over 8 sessions.

The “**Funambule, for balanced stress management” program,** developed by Dumont and his team (Dumont, 2012) is an intervention program designed to help young people aged from 12 to 18 to manage their stress better. It is undertaken once a week in 8 different sessions of at least an hour and a half each. The contents of the sessions were organised into 4 parts in order to target 4 goals: the perception of stress, the body, thought, and adaptation strategies. Its conception was inspired by Lazarus and Folkmans’s theory of 1984, which aimed to strengthen protective resources. Beneficial effects and a significant improvement of stress management in the experimental group were noted for this program (Dumont, 2012).

The “I’m managing my stress” program we’ve elaborated has the same objectives and aims, but it is a pilot program inspired by “Funambule”. Nevertheless, we have adapted it to fit the Internet and our studied population (adults, not children in like the original program). The program is made up of four sessions, 20 minutes long each, including psycho-education, practical exercises and one to two weekly activities that the participant is asked to complete (prescription of tasks, as is customary in cognitive-behavioural techniques). The goal is for students to learn easy techniques to help them face stressful situations in a better way.

**The themes of stress management interverntion via the Internet:**

- The first session is “ psycho-education” with information so the participants can spot and understand stress, measure its level, and determine its sources.
- The second session aims to work on body relaxation with practical exercises adapted to the Internet such as the contraction and relaxation method or breathing into a balloon.
- The third session is based on cognitive behavioural techniques, such as Beck’s three columns, the Meichenbaum method and problem-solving to work on thinking.
- The fourth session focuses on adaptation strategies such as time management, preparation for a test and the use of anti-stress cards.

**References:**

Adlaf, E. M., Gliksman, L., Demers, A., & Newton-Taylor, B. (2001). The Prevalence of Elevated Psychological Distress Among Canadian Undergraduates: Findings from the 1998 Canadian Campus Survey. *Journal of American College Health*, *50*(2), 67–72. https://doi.org/10.1080/07448480109596009

Baykan, Z., Naçar, M., & Cetinkaya, F. (2012). Depression, anxiety, and stress among last-year students at Erciyes University Medical School. *Academic Psychiatry: The Journal Of The American Association Of Directors Of Psychiatric Residency Training And The Association For Academic Psychiatry*, *36*(1), 64–65. https://doi.org/10.1176/appi.ap.11060125

Bayram, N., & Bilgel, N. (2008). The prevalence and socio-demographic correlations of depression, anxiety and stress among a group of university students. *Social Psychiatry and Psychiatric Epidemiology*, *43*(8), 667–672. https://doi.org/10.1007/s00127-008-0345-x

Bewick, B., Koutsopoulou, G., Miles, J., Slaa, E., & Barkham, M. (2010). Changes in undergraduate students’ psychological well‐being as they progress through university. *Studies in Higher Education*, *35*(6), 633–645. https://doi.org/10.1080/03075070903216643

Bolognini, M., Bettschart, W., Zehnder-Gubler, M., & Rossier, L. (1989). The validity of the French version of the GHQ-28 and PSYDIS in a community sample of 20 year olds in Switzerland. *European Archives of Psychiatry and Neurological Sciences*, *238*(3), 161–168. https://doi.org/10.1007/BF00451005

Boujut, E., Koleck, M., Bruchon-Schweitzer, M., & Bourgeois, M. L. (2009). Mental health among students: A study among a cohort of freshmen. *Annales Médico-Psychologiques*, *167*(9), 662–668. https://doi.org/10.1016/j.amp.2008.05.020

Bughi, S. A., Sumcad, J., & Bughi, S. (2006). Effect of brief behavioral intervention program in managing stress in medical students from two southern California universities. *Medical Education Online*, *11*, 1–8.

Chabrol, H., Carlin, E., Michaud, C., Rey, A., Cassan, D., Juillot, M., … Callahan, S. (2004). Étude de l’échelle d’estime de soi de Rosenberg dans un échantillon de lycéens. *Neuropsychiatrie de l’Enfance et de l’Adolescence*, *52*(8), 533–536. https://doi.org/10.1016/j.neurenf.2004.09.007

Chiauzzi, E., Brevard, J., Thurn, C., Decembrele, S., & Lord, S. (2008). MyStudentBody–Stress: An online stress management intervention for college students. *Journal of Health Communication*, *13*(6), 555–572. https://doi.org/10.1080/10810730802281668

Collange, J., Bellinghausen, L., Chappé, J., Saunder, L., & Albert, E. (2013). Stress perçu : à partir de quel seuil devient-il un facteur de risque pour les troubles anxiodépressifs ? *Archives Des Maladies Professionnelles et de l’Environnement*, *74*(1), 7–15. https://doi.org/10.1016/j.admp.2012.12.009

university. *BMC Psychiatry*, *15*, 26. https://doi.org/10.1186/s12888-015-0403-3

Dahlin, M., Joneborg, N., & Runeson, B. (2005). Stress and depression among medical students: a cross-sectional study. *Medical Education*, *39*(6), 594–604. https://doi.org/10.1111/j.1365-2929.2005.02176.x

Deasy, C., Coughlan, B., Pironom, J., Jourdan, D., & Mannix-McNamara, P. (2014). Psychological Distress and Coping amongst Higher Education Students: A Mixed Method Enquiry. *PLOS ONE*, *9*(12), e115193. https://doi.org/10.1371/journal.pone.0115193

Dumont, M. (2012). *Funambule Pour une gestion équilibrée du stress*. Québec: Septembre éditeur. Retrieved from http://www.septembre.com/livres/funambule-1259.html

Dyrbye, L. N., Harper, W., Durning, S. J., Moutier, C., Thomas, M. R., Massie, F. S., Jr., … Shanafelt, T. D. (2011). Patterns of distress in US medical students. *Medical Teacher*, *33*(10), 834–839. https://doi.org/10.3109/0142159x.2010.531158

Furr, S. R., Westefeld, J. S., McConnell, G. N., & Jenkins, J. M. (2001). Suicide and depression among college students: A decade later. *Professional Psychology: Research and Practice*, *32*(1), 97. https://doi.org/10.1037/0735-7028.32.1.97

George, D. R., Dellasega, C., Whitehead, M. M., & Bordon, A. (2013). Facebook-based stress management resources for first-year medical students: A multi-method evaluation. *Computers in Human Behavior*, *29*(3), 559–562. https://doi.org/10.1016/j.chb.2012.12.008

Goldberg, D. P., & Hillier, V. (1979). A scaled version of the General Health Questionnaire. *Psychological Medicine*, *9*(1), 139–145.

Grant, F., Guille, C., & Sen, S. (2013). Well-Being and the Risk of Depression under Stress. *PLOS ONE*, *8*(7), e67395. https://doi.org/10.1371/journal.pone.0067395

Humphris, G., Blinkhorn, A., Freeman, R., Gorter, R., Hoad-Reddick, G., Murtomaa, H., … Splieth, C. (2002). Psychological stress in undergraduate dental students: baseline results from seven European dental schools. *European Journal of Dental Education*, *6*(1), 22–29. https://doi.org/10.1034/j.1600-0579.2002.060105

Koochaki, G. M., Charkazi, A., Hasanzadeh, A., Saedani, M., Qorbani, M., & Marjani, A. (2011). Prevalence of stress among Iranian medical students: a questionnaire survey. *Eastern Mediterranean Health Journal*, *17*(7), 593–598.

Lavender, J. M., De Young, K. P., & Anderson, D. A. (2010). Eating Disorder Examination Questionnaire (EDE-Q): Norms for undergraduate men. *Eating Behaviors*, *11*(2), 119–121. https://doi.org/10.1016/j.eatbeh.2009.09.005

Luce, K. H., Crowther, J. H., & Pole, M. (2008). Eating Disorder Examination Questionnaire (EDE-Q): Norms for undergraduate women. *International Journal of Eating Disorders*, *41*(3), 273–276. https://doi.org/10.1002/eat.20504

Montero-Marín, J., Demarzo, M. M. P., Stapinski, L., Gili, M., & García-Campayo, J. (2014). Perceived Stress Latent Factors and the Burnout Subtypes: A Structural Model in Dental Students. *PLOS ONE*, *9*(6), e99765. https://doi.org/10.1371/journal.pone.0099765

Nerdrum, P., Rustøen, T., & Rønnestad, M. H. (2006). Student Psychological Distress: A psychometric study of 1750 Norwegian 1st‐year undergraduate students. *Scandinavian Journal of Educational Research*, *50*(1), 95–109. https://doi.org/10.1080/00313830500372075

O’Brien, L., Mathieson, K., Leafman, J., & Rice-Spearman, L. (2012). Level of stress and common coping strategies among physician assistant students. *The Journal Of Physician Assistant Education: The Official Journal Of The Physician Assistant Education Association*, *23*(4), 25–29. https://doi.org/10.1097/01367895-201223040-00006

Pilcher, J. J., Ginter, D. R., & Sadowsky, B. (1997). Sleep quality versus sleep quantity: Relationships between sleep and measures of health, well-being and sleepiness in college students. *Journal of Psychosomatic Research*, *42*(6), 583–596. https://doi.org/10.1016/S0022-3999(97)00004-4

Robotham, D., & Julian, C. (2006). Stress and the higher education student: a critical review of the literature. *Journal of Further and Higher Education*, *30*(2), 107–117. https://doi.org/10.1080/03098770600617513

Schulz, K. F., Altman, D. G., & Moher, D. (2010). CONSORT 2010 Statement: updated guidelines for reporting parallel group randomised trials. *BMJ*, *340*, c332. https://doi.org/10.1136/bmj.c332

Shamsuddin, K., Fadzil, F., Ismail, W. S. W., Shah, S. A., Omar, K., Muhammad, N. A., … Mahadevan, R. (2013). Correlates of depression, anxiety and stress among Malaysian university students. *Asian Journal Of Psychiatry*, *6*(4), 318–323. https://doi.org/10.1016/j.ajp.2013.01.014

Stallman, H. M. (2010). Psychological distress in university students: A comparison with general population data. *Australian Psychologist*, *45*(4), 249–257. https://doi.org/10.1080/00050067.2010.482109

Strenna, L., Chahraoui, K., & Vinay, A. (2009). Santé psychique chez les étudiants de première année d’école supérieure de commerce: liens avec le stress de l’orientation professionnelle, l’estime de soi et le coping. *L’orientation scolaire et professionnelle*, (38/2), 183–204. https://doi.org/10.4000/osp.1902

Vallerand, R. J., & Bissonnette, R. (1990). Construction et validation de l’Échelle de Satisfaction dans les Études (ESDE). [Construction and validation of the Scale of Satisfaction in Studies.]. *Canadian Journal of Behavioural Science/Revue Canadienne Des Sciences Du Comportement*, *22*(3), 295–306. https://doi.org/10.1037/h0078987

Vallieres, E. F., & Vallerand, R. J. (1990). Traduction et validation canadienne-française de l’échelle de l’estime de soi de Rosenberg. *International Journal of Psychology*, *25*(2), 305–316. https://doi.org/10.1080/00207599008247865

Vandentorren, S., Verret, C., Vignonde, M., & Maurice-Tison, S. (2005). Besoins d’information en santé des étudiants au service inter-universitaire de médecine préventive de Bordeaux. *Santé publique*, *17*(1), 47–56. https://doi.org/10.3917/spub.051.0047

Vliet, H. V., & Andrews, G. (2009). Internet-based course for the management of stress for junior high schools. *Australasian Psychiatry*, *43*(4), 305–309. https://doi.org/10.1080/00048670902721145

Walsh, J. M., Feeney, C., Hussey, J., & Donnellan, C. (2010). Sources of stress and psychological morbidity among undergraduate physiotherapy students. *Physiotherapy*, *96*(3), 206–212. https://doi.org/10.1016/j.physio.2010.01.005

Wong, J. G. W. S., Cheung, E. P. T., Chan, K. K. C., Ma, K. K. M., & Tang, S. W. (2006). Web-based survey of depression, anxiety and stress in first-year tertiary education students in Hong Kong. *The Australian And New Zealand Journal Of Psychiatry*, *40*(9), 777–782. https://doi.org/10.1111/j.1440-1614.2006.01883.x

Zetterqvist, K., Maanmies, J., Ström, L., & Andersson, G. (2003). Randomized controlled trial of Internet-based stress management. *Cognitive Behaviour Therapy*, *32*(3), 151–160. https://doi.org/10.1080/16506070310016248

Zivin, K., Eisenberg, D., Gollust, S. E., & Golberstein, E. (2009). Persistence of mental health problems and needs in a college student population. *Journal of Affective Disorders*, *117*(3), 180–185. https://doi.org/10.1016/j.jad.2009.01.001
