# Supplementary material for: Can we learn to manage stress? A randomized controlled trial carried out on university students
Source: PLoS One. 2018 Sep 5;13(9):e0200997. doi: 10.1371/journal.pone.0200997 (PMC6124718; doi:10.1371/journal.pone.0200997)
Supplement: S5 File — (PDF) [file pone.0200997.s005.pdf]

CNIL
8 rue de Vivienne - 75083 PARIS cedex 02
T. 01 53 73 22 22 - F. 01 53 73 22 00
www.cnil.fr

Madame SALEH Dalia
UNIVERSITE PARIS OUEST NANTERRE LA DEFENSE, L'UFR SPSE
200 AVENUE REPUBLIQUE, 92001 NANTERRE
92001 NANTERRE

RÉCÉPISSÉ

DÉCLARATION NORMALE

Numéro de déclaration
1811031 v 0

du 18 novembre 2014

A LIRE IMPERATIVEMENT
La délivrance de ce récépissé atteste que vous avez effectué une déclaration de votre traitement à la CNIL et que votre dossier est formellement complet. Vous pouvez mettre en œuvre votre traitement. Cependant, la CNIL peut à tout moment vérifier, par courrier ou par la voie d'un contrôle sur place, que ce traitement respecte l'ensemble des dispositions de la loi du 6 janvier 1978 modifiée en 2004. En tout état de cause, vous êtes tenu de respecter les obligations prévues par la loi et notamment :
1) La définition et le respect de la finalité du traitement,
2) La pertinence des données traitées,
3) La conservation pendant une durée limitée des données,
4) La sécurité et la confidentialité des données,
5) Le respect des droits des intéressés : information sur leur droit d'accès, de rectification et d'opposition.

Organisme déclarant

Nom : UNIVERSITE PARIS OUEST NANTERRE LA DEFENSE, L'UFR SPSE

Service :

Adresse : 200 AVENUE REPUBLIQUE, 92001 NANTERRE

Code postal : 92001
Ville : NANTERRE

N° SIREN ou SIRET :
199212044 00135

Code NAF ou APE :
8542Z

Tél. : 08 99 23 66 78
Fax. :

Traitement déclaré

Finalité : L'ENSEMBLE DE NOS RESULTATS (LE STRESS ET LA DETRESSE PSYCHOLOGIQUE SONT DES DIFFICULTES REPANDUES CHEZ LES ETUDIANTS UNIVERSITAIRES FRANCAIS) NOUS AMENE A CONSIDERER L'INTERET D'ADAPTER UNE INTERVENTION DE GESTION DU STRESS AUPRES DES ETUDIANTS A L'UNIVERSITE, TELLE QUE CELLE VALIDEE AU CANADA PAR MICHELLE DUMONT, QUE NOUS ADAPTONS ET METTONS EN PLACE SUR INTERNET DANS LA SECONDE ETAPE DE CE TRAVAIL. ELLE SE COMPOSE DE QUATRE SEANCES, CHACUNE COMPRENANT QUELQUES EXERCICES PRATIQUES ET UNE

Isabelle FALQUE PIERROTIN
Présidente
